# Pharmaceutical pricing, cost containment and new treatments for rare diseases in children

**DOI:** 10.1186/s13023-014-0152-2

**Published:** 2014-10-28

**Authors:** Peter Stella, Gabrielle Gold-von Simson

**Affiliations:** Department of Pediatrics, NYU Langone Hospital, New York, NY USA

**Keywords:** Orphan drugs, Drug pricing, Pediatrics, Affordable care act, 340b, Medicaid

## Abstract

Cost-containment in healthcare spending has become a central issue in public policy and healthcare reform, especially as the affordable care act adds millions of people to public and private insurance rolls. In this climate, longstanding criticism of pharmaceutical pricing has grown sharper, and many in both policy and medicine have characterized the costs of newly developed drugs as both exorbitant and wasteful of scarce healthcare resources. At the same time, pharmaceutical research and development pipeline costs are increasing exponentially.

Price resistance poses a significant threat to the development of drugs to treat rare pediatric diseases, where exceptionally high prices are a sine qua non of commercial viability. This article examines the trends in public discussion of high cost drugs and the potential consequences for orphan drug development. We conclude that despite growing public hostility towards high unit costs, drugs that treat rare diseases in children are likely to remain well-compensated and commercially viable.

## Background

**Figure 1:**
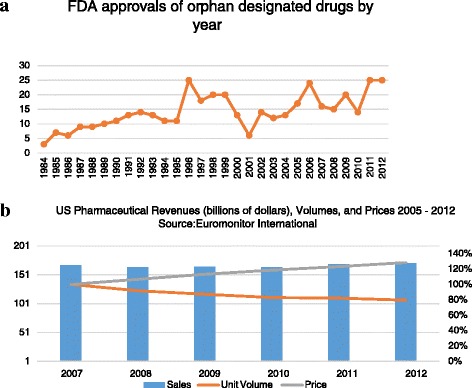
**Recent drug approvals, volumes, and prices.** (**a**. Trajectory of FDA Approval for Orphan Drugs, 1984-2012 [11]. **b**. Recent trends in pharmaceutical revenue, pricing and volume) [1].

Although total sales of pharmaceuticals in the United States were essentially flat from 2007 to 2012, this stable revenue conceals a marked shift in the industry, as drug companies have increasingly benefited from aggressive pricing despite declining volumes of drugs sold [1]. (Figure 1) This trend has many plausible causes, from routine changes in portfolio composition due to patent expirations of particularly successful mass market drugs, to evolving healthcare delivery and payment systems, and even fundamental improvements in basic science and biotechnology. But whatever the cause, this trend has significant political and public health implications, and therefore is of serious concern to those who discover, develop, manufacture, or recommend the use of very high cost drugs.

## Discussion

Drugs that cost hundreds of thousands of dollars per year make for sensational bullet points in debates over healthcare cost containment. Whereas other major cost drivers, such as dialysis or joint replacements, bring to mind images of ill people undergoing dangerous and complex therapies with the help of skilled professionals, it is much harder to justify the idea of a single pill, which needs only be swallowed, costing as much a person may make in a month. Put another way, expensive drugs are easy to criticize because the efforts expended to develop them are extremely costly, but these costs are hard for the casual observer to see. Additionally, for most well people who do not spend much of their time in the hospital or at doctors’ offices, pharmaceuticals are the most visible part of the healthcare system with which they interact commercially or financially.

In any case, the point remains that expensive drugs attract disproportionate attention and hostility in political and policy discussions. The negative press that this generates is particularly worrisome in the case of rare diseases that affect children, where high drug pricing, broad political support, and favorable regulatory attitudes have long been the bedrock of the economic “deal” between industry, government, and researchers underlying the development of therapies for previously untreatable “orphan” diseases. The prices for these drugs are, and always have been, very high.

The first drug approved for enzyme replacement therapy for a lysosomal storage disease, Genzyme’s Ceredase (Aglucerase), launched in 1991 with a price of approximately $150,000 per year (*in 1991 dollars)*. Prices of these magnitude are far from unusual in the fields of metabolic disease and genetics. Ceredase’s successor, Cerezyme currently costs approximately $200,000 for every year of therapy. Other drugs, in areas like pulmonology or immunology, can be even more expensive, as Vertex pharmaceuticals new CF treatment Ivacaftor (kalydeco) launched with a price of over $300,000 per year.

Despite a long history as the status quo, and a share of total healthcare spending that is estimated at only ~ 0.5% [2], the combination of public focus on cost containment and dramatic prices has begun to make even those directly responsible for the care of children with rare illnesses fear that expenditures on pharmaceuticals for rare disease will consume resources that could provide more patient benefit elsewhere [3-5].

However, despite these fears, regulatory commitment to high prices for orphan drugs remains strong, as can be seen in the recent debate over the United States Department of Health and Human Services’ (HHS) attempt to apply 340b pricing to orphan drugs under some circumstances. Although this attempt has been invalidated in a recent court case on the grounds that it represented an illegal extension of regulatory discretion into the legislative sphere, the specifics of HHS’s original intentions are illuminating when it comes to evaluating the prospects of very high priced drugs [6].

These drugs have relied favorable regulatory and legislative treatment that began with the Orphan Drug Act of 1983 (ODA), which was specifically designed to create the financial incentives for pharmaceutical companies to develop treatments for drugs outside of the traditional mass market, small molecule paradigm. It provides several incentives including a seven year market exclusivity window independent of patent protection, waiver of FDA fees, and tax breaks for drugs to treat conditions defined as rare, that is with a prevalence of under 200,000 people in the United States.

In contrast to this system of generous supports, the 340b program is an example of stern government price control. It requires drug companies to offer discounted drugs to “safety net” organizations, including HIV centers, hospitals with Medicaid heavy payor mixes, children’s hospitals, and others [7], although the laws governing the program explicitly excluded orphan drugs. The Affordable Care Act (ACA) mandated the expansion of this program to several new types of organization, and in July 2013, HHS released a ruling stating that 340b discounts should be applied to orphan drugs, when those drugs were used for conditions other than those for which they received orphan designations [8].

Although the idea of pushing industry to lower prices on orphan drugs might seem to indicate an erosion of support for the project of orphan drug development, the focus on indications is revealing of HHS’s likely goals. This is because while the “idea” of an orphan drug is a specific therapy directed a single rare disease, orphan status is given to a far wider range of agents, including those that are are used outside that designation for a variety of related conditions, which may be relatively common. This is particularly true in oncology where, many anti-neoplastics, such as Imatinib mesylate, (Gleevec®, Novartis Oncology), actually have multiple orphan designations. Overall, there are 1.5 orphan designations per approved drug, and as much as 90% of sales for drugs with orphan designations come outside of that orphan window [9]. Furthermore, many drugs have orphan designation only by virtue of relative narrow indications, such as for use as second or third line treatments, or in highly specific populations [10].

Taken in this context, HHS attempt to apply 340b pricing appears to be a very specific cost control measure, aimed at combatting the expanding use of the ODA by the pharmaceutical industry and to erode the status of drugs whose orphan status is based on a narrow indication or use in a subset of a more common disease.

In fact, what is remarkable here is that congress provided explicit protection of orphan drugs from drug discounting, and HHS, though clearly under pressure to undermine drug prices, showed no desire to place pressure on orphan indications, which in our reading signals an unusually solid commitment on the part of the regulatory and legislative community to protect the economic value of treatments for rare diseases, even in the face of mounting pressures on healthcare spending.

In the short term, there is no doubt that the Court’s ruling adds value to having an orphan designation in that it protects a drug from 340b discounting. As a consequence, we may expect an attempt by pharmaceutical companies to have their high priced “specialty” drugs so designated by financing new studies in orphan areas. However it is possible that HHS will appeal this ruling, or that the court’s decision could spur legislative action to ratify HHS’s interpretation of the rule. It is reasonable to ask what effects that a successful appeal or new legislation might have on the market for further orphan drug development. But even in such a case we see no indication in the treatment of this case that suggests any desire to create price pressure on drugs used for “real” orphan designations.

So while regulatory and legislative bodies are likely to continue to support incentives for drugs developed to treat “truly” rare disease, another threat looms- the willingness and ability of payors to continue to provide reimbursement at the very high (> $100,000/year) costs that is required to make these drugs economically viable for their developers. Assuming that payors behave as profit maximizing entities, their current high willingness to pay is driven by a simple calculation. While orphan drugs are strikingly expensive on a unit basis, the total volume of drug sold is so low that the impact on payors is minor, especially considering the reputational risk involved in denying disease-modifying treatment to pediatric patients. However, there is a perception among some that the number of these expensive drugs is increasing, and that the total cost of such drugs will continue to mount [11].

This perception, however, is more fear than fact. While it is true that there has been an increase in the number of drugs applying for orphan designations, drug approvals for these drugs have been essential flat since 2006. The overall number of annual FDA approvals for orphan drugs has not increased since 1996 [11]. (Figure 1b). When this is taken in concert with the previously mentioned issue of “narrow indication” orphan status, and the fact that very few of these orphan drugs are actually being used in children, the notion of an exploding number of treatments for rare pediatric disease is unfounded.

Other recent legislative changes should provide additional support for the development of therapies for rare pediatric diseases, such as the pediatric rare disease voucher program, which has provided vouchers offering priority review for an additional drug to firms who develop orphan drugs for orphan drugs which occur in those under 18 years of age. There are also provisions in the ACA or an accelerated approval program for “breakthrough drugs” that could be applied to most therapies for these diseases. Finally, the ACA mandates an increased role for patient stakeholders in FDA reviews, and in doing so, will place significant pressure on FDA to look favorably on these drugs, as rare disease and disease-specific patient advocacy working groups are quite savvy at leveraging power and forging pharmaceutical partnerships.

## Summary

Taken together, the variety of incentives created to encourage the development of new therapies rare pediatric diseases, and regulatory willingness to exclude these therapies even while attempting to impose cost containment on orphan designated drugs overall paints a reassuring picture for those who are concerned that unit price sensitivity will threaten the development of these therapies.

Cost pressures are undoubtedly a serious risk for the drug discovery and development industry, but the drugs used to treat rare pediatric diseases will likely remain a special, protected class, well insulated from the general climate. As such, pediatric rare and orphan drug development should continue to be an island of opportunity for industry, and a source of hope and solace for patients, families, and the community.
